# Unhealthy dietary behaviors and associated factors among adolescents: a territory-wide study

**DOI:** 10.1186/s12889-026-26935-y

**Published:** 2026-03-18

**Authors:** Claire Chenwen Zhong, Zhaojun Li, Mingtao Chen, Zehuan Yang, Vera M. W. Keung, Amelia Lo, Calvin Cheung, Junjie Huang, Martin C. S. Wong

**Affiliations:** 1 The Jockey Club School of Public Health and Primary Care, Faculty of Medicine, The Chinese University of Hong Kong, Hong Kong SAR, 999077 China; 2 Centre for Health Education and Health Promotion, Faculty of Medicine, The Chinese University of Hong Kong, Hong Kong SAR, China

**Keywords:** Unhealthy dietary behaviors, Adolescents, Dietary patterns, Health Literacy Measure for Adolescents, Mental Toughness Scale for Adolescents

## Abstract

**Background:**

Dietary behaviors during adolescence greatly influence lifelong health habits, with patterns and nutritional deficiencies at this stage potentially carrying into adulthood and affecting long-term health. This study examined associations between sociodemographic characteristics, health literacy, mental toughness, and unhealthy dietary behaviors among Hong Kong adolescents.

**Methods:**

A cross-sectional survey was conducted between September and November 2024 with secondary school students (Grades 1–4) from 20 schools in Hong Kong. Participants completed a self-administered questionnaire assessing sociodemographic variables, socioeconomic status, dietary behaviors, physical activity, health literacy, and mental toughness. Descriptive analysis of the factors was conducted to illustrate the frequency and percentage distributions. Both crude prevalence and weighted prevalence of unhealthy dietary behaviors were calculated. An survey-weighted logistic regression model was employed to examine the factors associated with unhealthy dietary habits.

**Results:**

A total of 1,423 secondary students were included in this study, comprising 802 females (56.4%) and 621 males (43.6%). The mean age of participants is 13.56 (standard deviation [SD]: ±1.39). Over half (51.0%) of adolescents skipped breakfast daily, while 86.1% and 83.8% reported inadequate vegetable and fruit consumption, respectively. In multivariable regression analysis, breakfast skipping was positively associated with excessive social media use (aOR = 1.668, 95% CI: 1.279–2.176, *p* < 0.001), and inversely related to sufficient sleep (aOR = 0.579, 95% CI: 0.447–0.751, *p* < 0.001), higher mental toughness(aOR = 0.692, 95% CI: 0.531–0.901, *p* = 0.006), and a higher level of health literacy(aOR = 0.659, 95% CI: 0.507–0.857, *p* = 0.002). Inadequate fruit intake was linked to older age groups (aOR_aged 13−14_= 1.497, 95%CI: 1.035–2.166, *p* = 0.032; aOR_aged>14_= 1.486, 95%CI:1.024–2.156, *p* = 0.037) and excessive screen time on game (aOR = 1.435, 95%CI:1.015–2.028,*p* = 0.041), while high socioeconomic status (aOR = 0.371, 95%CI: 0.213–0.643, *p* < 0.001) and higher level of health literacy (aOR = 0.674, 95% CI: 0.479–0.950, *p* = 0.024) reduced this risk. However, no significant association was found between factors and insufficient vegetable intake in the multivariable regression analysis.

**Conclusion:**

Unhealthy dietary behaviors are widespread among Hong Kong adolescents and are associated with modifiable behavioral, psychological, and sociodemographic factors. Targeted multi-level interventions focusing on health literacy, physical activity, and screen time regulation are recommended.

**Supplementary Information:**

The online version contains supplementary material available at 10.1186/s12889-026-26935-y.

## Introduction

Healthy dietary habits refer to the intake of nutrients in appropriate proportions to meet physiological needs without excessive intake [1]. Dietary behaviors during adolescence significantly impact the development of lifelong health habits, as patterns of behavior and underlying nutritional deficiencies at this stage may continue into adulthood, with long-term consequences for future health [2–4]. In recent years, unhealthy dietary behaviors among adolescents, such as skipping breakfast, high sugar consumption, and insufficient intake of fruits and vegetables, have been linked to various adverse health outcomes, particularly obesity and overweight, which have emerged as critical public health challenges [5, 6], in which the diseases associated with obesity, such as type II diabetes, coronary artery disease, and stroke, has increased the disease burden globally [7]. According to estimates by the World Health Organization (WHO), in 2022, more than 390 million children and adolescents aged 5–19 years were identified as overweight globally, accounting for 20% of the population in this age group [8]. Another study reported similar findings, stating that the prevalence of overweight in the adolescent population aged 12–15 years could be as high as 27.7%, with an observed rising trend [9]. In Hong Kong, concerns have been raised regarding the weight issues among adolescents. Several epidemiological studies indicate that the body mass index (BMI) of Hong Kong adolescents has been consistently rising over the past few decades [10–12]. Additionally, a survey conducted by the Hong Kong Student Health Service Centre reveals that the prevalence of overweight among secondary school students has reached 20.0%, with no significant improvement observed compared to previous years [13].

The association between unhealthy dietary behaviors and obesity has been well-established [6], the excessive intake of fructose and ultra-processed food consumption, like jam and potato chips, posed a higher risk of metabolic dysfunction-associated steatotic liver disease among children and adolescents [14]. In contrast, healthy dietary practices—such as adequate intake of fruits and vegetables and daily breakfast consumption—have been identified as protective factors against cardiovascular disease and mental health issues [15, 16]. Notably, a recent study has identified the positive effects of nutraceutical foods, such as extra virgin olive oil, in the context of childhood obesity, demonstrating their role in reducing oxidative stress and chronic inflammation associated with fat accumulation [17]. This highlights the critical importance of dietary component patterns in preventing obesity and its associated complications. More importantly, healthy dietary habits formed during this stage may help prevent chronic diseases such as cancer and diabetes in adulthood [18, 19]. These findings highlight the importance of health prevention through interventions aimed at improving adolescent dietary behaviors.

Over the past few decades, various authorities have attempted to establish guidelines and recommendations for healthy dietary behaviors among adolescents in order to mitigate the health risks associated with unhealthy eating habits. For instance, the WHO recommends that adolescents should consume 400 g (or five servings) of vegetables and fruits per day [20]. The Department of Health in Hong Kong suggests that adolescents should have at least three servings of vegetables and two servings of fruits daily [21]. Similar recommendations are made by authorities in the U.S., Australia, and China [22–24]. Nevertheless, the dietary behaviors of adolescents remain unsatisfactory. A study involving 193,388 adolescents across 31 countries found that approximately 75% of adolescents reported insufficient daily consumption of fruit and vegetables [25]. Research from the United States indicates that more than 90% of adolescents do not meet the recommended intake of fruits and vegetables [26], similar to findings from Hong Kong [27]. The Hong Kong Population Health Survey 2020-22 estimated that 96.9% of adolescents and younger adults aged 15–24 had an average of less than 3 servings of vegetables per day [27]. Recent evidence from the Student Health Services Centre further corroborates this observation, demonstrating that as many as 97.0% of secondary school students do not meet the recommended intake of vegetables and fruits [13].

Given this situation, scholars have made significant efforts to intervene early in unhealthy dietary behaviors among adolescents. However, these interventions are not always effective [28, 29]. Although evidence suggested that interventions in dietary behaviours, particularly in school-based settings, hold potential, their effectiveness, especially long-term efficacy, is commonly constrained by adherence among adolescents [29]. An intervention study involving 3,941 children and adolescents indicated that while cluster intervention designs are beneficial, their effectiveness is inconsistent across groups: significant improvement in dietary habits was observed among adolescent females, but not among adolescent males [30]. The socio-ecological model developed by Bronfenbrenner emphasizes that the establishment and decision-making of health behaviors are complex, not only on the interconnectedness of interpersonal, organizational, community, and policy levels, but also involving individual-level factors such as the socio-demographic characteristics [31]. The significance of individual attributes, including economic feasibility and personal preferences, has been well recognized in the adoption of healthy dietary practices [28, 32]. Therefore, it is imperative to develop strategies that facilitate tailored interventions aimed at promoting healthy dietary habits among at-risk populations most in need, with the objective of maximizing the effectiveness of these interventions through the enhancement of adherence [33].

However, to the best of our knowledge, limited studies have assessed the relationship between individual-level characteristics of adolescents and unhealthy dietary behaviors. Existing studies primarily focus on the health outcomes of unhealthy eating habits or the influence of external factors [34, 35]. Therefore, this study aims to identify the potential factors contributing to unhealthy dietary behaviours among adolescents in Hong Kong, with a specific focus on examining the association between individual-level characteristics and unhealthy dietary behaviours, including skipping breakfast, insufficient vegetable intake, and insufficient fruit intake, thereby identifying at-risk subgroups to facilitate the development of more tailored and effective interventions and policies. Additionally, the study seeks to address the following questions: What are the individual-level factors associated with unhealthy dietary habits among adolescents in Hong Kong? and Who are at a higher risk of engaging in unhealthy dietary habits?

## Methods

### Study design and participants

This is a cross-sectional study investigating factors associated with dietary habits among adolescents in Hong Kong, conducted between September and November 2024. The study was designed and reported in accordance with the Strengthening the Reporting of Observational Studies in Epidemiology guidelines [36]. An invitation was sent out to all schools participating in the “Health Education Built-on Project” carried out by the Centre for Health Education and Health Promotion of the Chinese University of Hong Kong. All students in grades 1 to 4 of secondary schools in Hong Kong were considered potentially eligible participants for this study. In Hong Kong’s education system, secondary students in grades 1 to 4 are typically aged 12 to 16 [37].

### Data collection

A total of 20 schools participating in the “Health Education Built-on Project” and were invited to participate in this research via email to provide detailed information about the study and its purpose. The invited schools are in 11 out of the 18 clusters in Hong Kong, providing a representative sample of adolescents across different clusters. After obtaining consent from the schools, hard copies of the questionnaires were sent to all participating schools. Students who agreed to participate in the study were asked to complete a self-administered questionnaire in the classroom, which took approximately 20–25 min. Trained teachers were responsible for overseeing the process. A total of 2,139 adolescents were approached to participate in the study, and 1,826 adolescents completed the questionnaire, resulting in a response rate of 85.37%. No incentive was provided for participation.

### Ethical consideration

Prior to the administration of the questionnaire, consent was obtained from both students and their parents participating in this study. Participants were informed about the voluntary and anonymous nature of the research. All completed questionnaires were secured in sealed envelopes and returned to the research office without access by teachers. The collected data was anonymized to ensure that no personally identifiable information was disclosed and was stored on a computer accessible exclusively to the researcher.

### Survey instrument

To capture individual-level characteristics potentially influencing unhealthy dietary behaviors among adolescents, a structured survey was administered, comprising four major sections. In addition, the scales employed in this study were translated into Traditional Chinese through a forward-backward translation process conducted by the research team to ensure accuracy. Both the English and the Traditional Chinese versions of the questionnaire were available for participants.

The first section collected socio-demographic information, including age, sex, and SES. SES was measured using the Family Affluence Scale II (FAS II), which included questions about whether the respondent had a private bedroom, the number of cars owned by the family, the number of household computers, and travel frequency over the past year. A composite score ranging from 0 to 7 was calculated and categorized into low (0–3), medium (4, 5), and high (6, 7) affluence levels [38]. The Cronbach’s alpha for the FAS II used in this study was 0.34.

The second section evaluated health-related behaviors through a self-designed questionnaire. The measurement of key covariates for health-related behavior were adapted from the Global School-based Student Health Survey (GSHS) developed by the WHO [39]. Items in this section assessed physical activity (including moderate- to vigorous-intensity activities without differentiating between school-based and extracurricular activities) over the past seven days, screen time on weekdays (including time spent on video games, electronic devices, and social media), average sleep duration, smoking and alcohol use in the past 30 days, and self-perceived weight status. Responses to these items provided information on modifiable lifestyle factors that might be associated with unhealthy dietary behaviors.

The third section assessed health literacy using the Health Literacy Measure for Adolescents (HELMA), a validated 44-item instrument covering eight dimensions: access to health information, reading ability, understanding, appraisal, utilization, communication, self-efficacy, and numeracy [40, 41]. Each item was scored on a 5-point rating scale, with higher scores indicating better health literacy [41]. The Cronbach’s alpha for the HELMA used in this study was 0.96.

The fourth section measured mental toughness using the Mental Toughness Scale for Adolescents (MTS-A), which consists of 18 items scored on a 4-point scale (from strongly disagree to strongly agree) [42]. The scale captures six psychological dimensions, namely challenge, confidence in interpersonal relationships, confidence in ability, emotional control, life control, and commitment. The Cronbach’s alpha for the MTS-A used in this study was 0.87.

### Outcome variables

The primary outcomes of this study were three specific unhealthy dietary behaviors: skipping breakfast, insufficient vegetable intake, and insufficient fruit intake. Skipping breakfast was defined as not consuming breakfast every day during the past week. Insufficient vegetable intake and insufficient fruit intake refer to the consumption of fewer than 1.5 bowls (i.e., three servings) of vegetables per day and consumption of fewer than 2 servings of fruit per day, respectively, according to the recommendation from the Hong Kong Centre for Health Protection [43].

### Data analysis

Descriptive statistics were computed and presented using frequencies and percentages for each factor. Besides crude prevalence of outcome variables, a weighted prevalence was used to estimate and represent the prevalence among the adolescent population in Hong Kong in 2024 (Supplementary Table 1) to strengthen the robustness of prevalence estimation. Group differences in dietary outcomes were examined using chi-square tests for categorical variables and independent sample t-tests for continuous variables. Survey-weighted univariable and multivariable logistic regression analyses using the Taylor linearization were performed to assess associations between individual-level factors and each of the three dietary outcomes: skipping breakfast, insufficient vegetable intake, and insufficient fruit intake, respectively. The following variables were included in the regression analysis, including socio-demographic factors (age, sex, and socioeconomic status), health-related behaviors (smoking, alcohol consumption, physical activity, sleep duration, screen time, and self-perceived weight status), as well as psychological and cognitive variables (mental toughness and health literacy). Variables were categorized as follows: age (< 13, 13–14, > 14 years), FAS status (low: 0–3; medium: 4–5; high: 6–7), recent smoking and alcohol use (past 30 days: yes/no), sufficient physical activity (≥ 1 h of moderate-to-vigorous activity daily), sufficient sleep (≥ 8 h per night), excessive screen time (more than 2 h per weekday on video games, electronic devices, or social media), and self-reported overweight status (“very overweight” or “slightly overweight”). Health literacy was dichotomized into low (score 44–144) and high (145–220) based on the median HELMA scores. Both crude odds ratios (ORs) and adjusted odds ratios (AORs) with 95% confidence intervals (CIs) were reported. A p-value of < 0.05 was considered statistically significant and indicated an association between the individual factor and unhealthy dietary behaviors. Missing data were addressed through a complete case analysis approach, whereby subjects with missing data were excluded from subsequent analyses. Two additional multivariable logistic regression models were provided as sensitivity analyses: one employed an ordinal logistic regression approach, and the other included only variables with *p* < 0.05 in the univariable logistic regression analysis. Data analyses were conducted using R version 4.2.1.

## Results

### Participant characteristics

Of the 1,826 surveys collected, a total of 1,423 validated surveys were included in this study after excluding those with missing data, comprising 802 females (56.4%) and 621 males (43.6%), with a mean age of 13.56 (SD:1.39). Among them, most participants were under the age of 13 (42.4%, *n* = 604), while 28.5% (*n* = 405) of participants were aged 13–14, and 29.1% (*n* = 414) were aged 15 or older. Approximately half were categorized as having medium socioeconomic status (SES) (49.0%, *n* = 697). The majority reported no smoking (98.4%, *n* = 1,400) and no alcohol consumption (91.6%, *n* = 1,314) in the past 30 days. In terms of health behaviors, 87.1% (*n* = 1,239) reported engaging in less than one hour of moderate-to-vigorous physical activity per day, and over half (55.7%, *n* = 792) reported insufficient sleep (< 8 h per day). Excessive screen time was also common, with 58.2% (*n* = 828) reporting more than two hours per day spent watching videos, 52.0% (*n* = 740) spending more than two hours daily on electronic games, and 51.4% (*n* = 732) devoting over two hours to social media. Additionally, 55.1% (*n* = 784) of participants were categorized as having low mental toughness, and 51.1% (*n* = 727) were identified as having low health literacy. Details could be found in Tables 1, 2 and 3.

**Table 1:** Participant characteristics by breakfast habit

	Overall	Having breakfast every day = Yes	Having breakfast every day = No	*p*
*n*	1,423	697 (49.0%)	726 (51.0%)	
Age				
Mean (SD)	13.56 (1.39)	13.46 (1.38)	13.66 (1.39)	
Age group				**0.010**
< 13	604 (42.4%)	324 (53.6%)	280 (46.4%)	
13–14	405 (28.5%)	184 (45.4%)	221 (54.6%)	
> 14	414 (29.1%)	189 (45.7%)	225 (54.3%)	
Sex				**0.001**
Female	802 (56.4%)	361 (45.0%)	441 (55.0%)	
Male	621 (43.6%)	336 (54.1%)	285 (45.9%)	
SES [0–9]				0.248
Low [0–2]	236 (16.6%)	116 (49.2%)	120 (50.8%)	
Mid [3–5]	697 (49.0%)	327 (46.9%)	370 (53.1%)	
High [6–9]	490 (34.4%)	254 (51.8%)	236 (48.2%)	
Smoking				0.245
No	1,400 (98.4%)	689 (49.2%)	711 (50.8%)	
Yes	23 (1.6%)	8 (34.8%)	15 (65.2%)	
Alcohol drinking				**0.008**
No	1,304 (91.6%)	653 (50.5%)	651 (49.5%)	
Yes	119 (8.4%)	44 (37.0%)	75 (63.0%)	
Sufficient physical activity				**0.035**
No	1,239 (87.1%)	593 (47.9%)	646 (52.1%)	
Yes	184 (12.9%)	104 (56.5%)	80 (43.5%)	
Sufficient sleep				**< 0.001**
No	792 (55.7%)	328 (41.4%)	464 (58.6%)	
Yes	631 (44.3%)	369 (58.5%)	262 (41.5%)	
Excessive screening time on video				**0.018**
No	595 (41.8%)	314 (52.8%)	281 (47.2%)	
Yes	828 (58.2%)	383 (46.3%)	445 (53.7%)	
Excessive screening time on electronic game				**< 0.001**
No	683 (48.0%)	371 (54.3%)	312 (45.7%)	
Yes	740 (52.0%)	326 (44.1%)	414 (55.9%)	
Excessive screening time on social media				**< 0.001**
No	691 (48.6%)	397 (57.5%)	294 (42.5%)	
Yes	732 (51.4%)	300 (41.0%)	432 (59.0%)	
Self-reported obesity				**0.002**
No	943 (66.3%)	490 (52.0%)	453 (48.0%)	
Yes	480 (33.7%)	207 (43.1%)	273 (56.9%)	
MTS-A				**< 0.001**
Low	784 (55.1%)	329 (42.0%)	455 (58.0%)	
High	639 (44.9%)	368 (57.6%)	271 (42.4%)	
Health literacy level				**< 0.001**
Low	727 (51.1%)	313 (43.1%)	414 (56.9%)	
High	696 (48.9%)	384 (55.2%)	312 (44.8%)	

*SES* Socioeconomic Status

Sufficient physical activity: Moderate/Vigorous exercise > = 1 h everyday; Sufficient sleep: Sleep at least 8 h; *MTS-A* Mental Toughness Scale for Adolescents, the scale [18–72] is composed of six dimensions, including challenge [3–12], commitment [3–12], emotion control [3–12], life control [3–12], confidence in abilities [3–12], and interpersonal confidence [3–12], with a higher score indicating higher mental toughness; Health literacy level: Scores for evaluating personal competencies for the access to, understanding of, appraisal of and application of health information in order to make sound decisions in everyday life; The prevalence refers to crude prevalence rate in this table

Significant values are shown in bold

**Table 2:** Participant characteristics by vegetable intake

	Overall	Sufficient vegetable intake	Insufficient vegetable intake	*p*
*n*	1,423	198 (13.9%)	1,225 (86.1%)	
Age				
Mean (SD)	13.56 (1.39)	13.59 (1.41)	13.56 (1.39)	
Age group				0.416
< 13	604 (42.4%)	86 (14.2%)	518 (85.8%)	
13–14	405 (28.5%)	49 (12.1%)	356 (87.9%)	
> 14	414 (29.1%)	63 (15.2%)	351 (84.8%)	
Sex				0.066
Female	802 (56.4%)	124 (15.5%)	678 (84.5%)	
Male	621 (43.6%)	74 (11.9%)	547 (88.1%)	
SES [0–9]				0.219
Low [0–2]	236 (16.6%)	27 (11.4%)	209 (88.6%)	
Mid [3–5]	697 (49.0%)	93 (13.3%)	604 (86.7%)	
High [6–9]	490 (34.4%)	78 (15.9%)	412 (84.1%)	
Smoking				0.430
No	1,400 (98.4%)	193 (13.8%)	1,207 (86.2%)	
Yes	23 (1.6%)	5 (21.7%)	18 (78.3%)	
Alcohol drinking				0.171
No	1,304 (91.6%)	176 (13.5%)	1,128 (86.5%)	
Yes	119 (8.4%)	22 (18.5%)	97 (81.5%)	
Sufficient physical activity				**0.007**
No	1,239 (87.1%)	160 (12.9%)	1,079 (87.1%)	
Yes	184 (12.9%)	38 (20.7%)	146 (79.3%)	
Sufficient sleep				0.302
No	792 (55.7%)	103 (13.0%)	689 (87.0%)	
Yes	631 (44.3%)	95 (15.0%)	536 (85.0%)	
Excessive screening time on video				0.231
No	595 (41.8%)	91 (15.3%)	504 (84.7%)	
Yes	828 (58.2%)	107 (12.9%)	721 (87.1%)	
Excessive screening time on electronic game				0.109
No	683 (48.0%)	106 (15.5%)	577 (84.5%)	
Yes	740 (52.0%)	92 (12.4%)	648 (87.6%)	
Excessive screening time on social media				0.801
No	691 (48.6%)	94 (13.6%)	597 (86.4%)	
Yes	732 (51.4%)	104 (14.2%)	628 (85.8%)	
Self-reported obesity				0.277
No	943 (66.3%)	144 (15.3%)	799 (84.7%)	
Yes	480 (33.7%)	54 (11.2%)	426 (88.8%)	
MTS-A				0.074
Low	784 (55.1%)	97 (12.4%)	687 (87.6%)	
High	639 (44.9%)	101 (15.8%)	538 (84.2%)	
Health literacy level				**0.004**
Low	727 (51.1%)	82 (11.3%)	645 (88.7%)	
High	696 (48.9%)	116 (16.7%)	580 (83.3%)	

Sufficient vegetable intake: Had >= 1.5 bowls of vegetables everyday; Insufficient vegetable intake: Had < 1.5 bowls of vegetables everyday; SES: Socioeconomic Status; Sufficient physical activity: Moderate/Vigorous exercise > = 1 h everyday; Sufficient sleep: Sleep at least 8 h; MTS-A: Mental Toughness Scale for Adolescents, the scale [18–72] is composed of six dimensions, including challenge [3–12], commitment [3–12], emotion control [3–12], life control [3–12], confidence in abilities [3–12], and interpersonal confidence [3–12], with a higher score indicating higher mental toughness; Health literacy level: Scores for evaluating personal competencies for the access to, understanding of, appraisal of and application of health information in order to make sound decisions in everyday life; The prevalence refers to crude prevalence rate in this table

Significant values are shown in bold

**Table 3:** Participant characteristics by fruit intake

	Overall	Sufficient fruit intake	Insufficient fruit intake	*p*
*n*	1,423	230 (16.2%)	1,193 (83.8%)	
Age				
Mean (SD)	13.56 (1.39)	13.31 (1.39)	13.61 (1.38)	
Age group				**0.002**
< 13	604 (42.4%)	122 (20.2%)	482 (79.8%)	
13–14	405 (28.5%)	55 (13.6%)	350 (86.4%)	
> 14	414 (29.1%)	53 (12.8%)	361 (87.2%)	
Sex				0.985
Female	802 (56.4%)	129 (16.1%)	673 (83.9%)	
Male	621 (43.6%)	101 (16.3%)	520 (83.7%)	
SES [0–9]				**< 0.001**
Low [0–2]	236 (16.6%)	24 (10.2%)	212 (89.8%)	
Mid [3–5]	697 (49.0%)	93 (13.3%)	604 (86.7%)	
High [6–9]	490 (34.4%)	113 (23.1%)	377 (76.9%)	
Smoking				0.487
No	1,400 (98.4%)	228 (16.3%)	1,172 (83.7%)	
Yes	23 (1.6%)	2 (8.7%)	21 (91.3%)	
Alcohol drinking				0.218
No	1,304 (91.6%)	216 (16.6%)	1,088 (83.4%)	
Yes	119 (8.4%)	14 (11.8%)	105 (88.2%)	
Sufficient physical activity				**0.021**
No	1,239 (87.1%)	189 (15.3%)	1,050 (84.7%)	
Yes	184 (12.9%)	41 (22.3%)	143 (77.7%)	
Sufficient sleep				0.217
No	792 (55.7%)	119 (15.0%)	673 (85.0%)	
Yes	631 (44.3%)	111 (17.6%)	520 (82.4%)	
Excessive screening time on video				0.355
No	595 (41.8%)	103 (17.3%)	492 (82.7%)	
Yes	828 (58.2%)	127 (15.3%)	701 (84.7%)	
Excessive screening time on electronic game				**0.001**
No	683 (48.0%)	133 (19.5%)	550 (80.5%)	
Yes	740 (52.0%)	97 (13.1%)	643 (86.9%)	
Excessive screening time on social media				0.204
No	691 (48.6%)	177 (25.6%)	514 (74.4%)	
Yes	732 (51.4%)	109 (14.9%)	623 (85.1%)	
Self-reported obesity				0.869
No	943 (66.3%)	154 (16.3%)	789 (83.7%)	
Yes	480 (33.7%)	76 (15.8%)	404 (84.2%)	
MTS-A				0.056
Low	784 (58.2%)	113 (14.4%)	671 (85.6%)	
High	639 (44.9%)	117 (18.3%)	522 (81.7%)	
Health literacy level				**0.009**
Low	727 (51.1%)	162 (22.3%)	565 (77.7%)	
High	696 (48.9%)	131 (18.8%)	565 (81.2%)	

Sufficient fruit intake: Had >= 2 servings of fruit everyday; Insufficient fruit intake: Had < 2 servings of fruit everyday; *SES* Socioeconomic Status; Sufficient physical activity: Moderate/Vigorous exercise > = 1 h everyday; Sufficient sleep: Sleep at least 8 h; MTS-A: Mental Toughness Scale for Adolescents, the scale [18–72] is composed of six dimensions, including challenge [3–12], commitment [3–12], emotion control [3–12], life control [3–12], confidence in abilities [3–12], and interpersonal confidence [3–12], with a higher score indicating higher mental toughness; Health literacy level: Scores for evaluating personal competencies for the access to, understanding of, appraisal of and application of health information in order to make sound decisions in everyday life; The prevalence refers to crude prevalence rate in this table

Significant values are shown in bold

### Distribution of dietary behaviors across participant characteristics

The prevalence of unhealthy dietary behaviors is presented in Tables 1, 2 and 3, with 51.0% of participants reporting skipping breakfast, 86.1% consuming insufficient vegetables, and 83.8% consuming insufficient fruit as recommended. For the prevalence rates of unhealthy dietary behaviors after adjusting for survey weights, see Supplementary Table 2. The results show similar prevalence rates to those in the primary analysis, estimating that approximately 53.1%, 85.7%, and 84.9% of adolescents skip breakfast, consume insufficient vegetables, and consume inadequate fruit, respectively.

As shown in Table 1 and Supplementary Fig. 1, 49.0% (*n* = 697) of participants reported having breakfast daily, whereas 51.0% (*n* = 726) did not. Chi-square tests revealed statistically significant differences in breakfast consumption across various subgroups. Participants aged 13–14 years, females, those who reported alcohol use, those with insufficient physical activity or sleep, and those who reported excessive screen time on video, electronic games, and social media were more likely to report not consuming breakfast daily (*p* < 0.05). Additionally, non-daily breakfast consumption was more frequently reported among participants who self-identified as overweight or obese, and among those with lower levels of mental toughness and health literacy.

Regarding vegetable intake (Table 2; Supplementary Fig. 2), only 13.9% (*n* = 198) of participants met the recommended daily intake, while 86.1% (*n* = 1,225) did not. The distribution of vegetable intake varied significantly by level of physical activity and health literacy, with participants reporting insufficient physical activity and lower health literacy more frequently falling into the group with inadequate vegetable consumption (*p* = 0.007 and *p* = 0.004, respectively).

Similar patterns were observed for fruit intake (Table 3; Supplementary Fig. 3). Only 16.2% (*n* = 230) of participants consumed an adequate amount of fruit, while 83.8% (*n* = 1,193) did not. The distribution of fruit intake significantly differed by age, socioeconomic status (SES), physical activity level, screen time on electronic games, and health literacy. Inadequate fruit intake was more commonly reported among those older than 14 years, those with lower SES, those engaging in less physical activity, those with excessive screen time on electronic games, and those with lower levels of health literacy (*p* < 0.05).

Supplementary Table 3 presents further analysis of participants who exhibited all three types of unhealthy dietary habits (40.3%; *n* = 573) and those who demonstrated only one or two types of unhealthy dietary habits (59.7%; *n* = 850). Adolescent exhibiting all three types of unhealthy dietary habits was more prevalent among adolescents who were older than 14 years, female, those with insufficient sleep duration, those who spent over two hours daily on screens for video/electronic games/social media, those who had a lower level of mental toughness, and a lower level of health literacy (*p* < 0.05).

### Factors associated with unhealthy dietary behaviors

The survey-weighted univariable and multivariable logistic regression analyses examining factors related to skipping breakfast, insufficient vegetable intake, and insufficient fruit intake are presented in Tables 4, 5 and 6. The multivariable results are summarized below.

**Table 4:** Factors associated with skipping breakfast (*N* = 1,423)

	Crude odds ratio (95% CI)	*P*-value	Adjusted odds ratio (95% CI)	*P*-value
Age group				
< 13	reference		reference	
13–14	1.365 (1.046–1.779)	**0.022**	1.259 (0.950–1.668)	0.109
> 14	1.325 (1.020–1.723)	**0.035**	1.109 (0.838–1.470)	0.469
Gender				
Female	reference		reference	
Male	0.704 (0.553–0.896)	**0.004**	0.800 (0.614–1.042)	0.098
SES				
Low	reference		reference	
Medium	1.179 (0.838–1.660)	0.344	1.200 (0.844–1.706)	0.309
High	0.847 (0.591–1.214)	0.365	0.877 (0.601–1.280)	0.496
Smoking				
No	reference		reference	
Yes	2.736 (1.063–7.042)	**0.037**	2.063 (0.723–5.889)	0.176
Alcohol drinking				
No	reference		reference	
Yes	1.875 (1.215–2.891)	**0.005**	1.309 (0.813–2.107)	0.267
Sufficient physical activity				
No	reference		reference	
Yes	0.620 (0.434–0.885)	**0.009**	0.735 (0.507–1.066)	0.104
Sufficient sleep				
No	reference		reference	
Yes	0.491 (0.385–0.626)	**< 0.001**	0.579 (0.447–0.751)	**< 0.001**
Excessive screening time on video				
No	reference		reference	
Yes	1.197 (0.939–1.525)	0.146	0.912 (0.700-1.189)	0.496
Excessive screening time on electronic game				
No	reference		reference	
Yes	1.536 (1.210–1.951)	**< 0.001**	1.288 (0.986–1.682)	0.063
Excessive screening time on social media				
No	reference		reference	
Yes	2.031 (1.600–2.580)	**< 0.001**	1.668 (1.279–2.176)	**< 0.001**
Self-reported obesity				
No	reference		reference	
Yes	1.303 (1.011–1.680)	**0.041**	1.156 (0.891–1.501)	0.275
MTS-A				
Low	reference		reference	
High	0.507 (0.397–0.646)	**< 0.001**	0.692 (0.531–0.901)	**0.006**
Health literacy level				
Low	reference		reference	
High	0.575 (0.452–0.731)	**< 0.001**	0.659 (0.507–0.857)	**0.002**

*SES* Socioeconomic Status

Sufficient physical activity: Moderate/Vigorous exercise > = 1 h everyday; Sufficient sleep: Sleep at least 8 h; MTS-A: Mental Toughness Scale for Adolescents, the scale [18–72] is composed of six dimensions, including challenge [3–12], commitment [3–12], emotion control [3–12], life control [3–12], confidence in abilities [3–12], and interpersonal confidence [3–12], with a higher score indicating higher mental toughness; Health literacy level: Scores for evaluating personal competencies for the access to, understanding of, appraisal of and application of health information in order to make sound decisions in everyday life

Significant values are shown in bold

**Table 5:** Factors associated with insufficient vegetable intake (*N* = 1,423)

	Crude odds ratio (95% CI)	*P*-value	Adjusted odds ratio (95% CI)	*P*-value
Age group				
< 13	reference		reference	
13–14	1.138 (0.767–1.690)	0.521	1.132 (0.761–1.684)	0.540
> 14	0.872 (0.606–1.255)	0.461	0.887 (0.604–1.303)	0.542
Gender				
Female	reference		reference	
Male	1.046 (0.735–1.490)	0.802	1.098 (0.753-1.600)	0.628
SES				
Low	reference		reference	
Medium	0.946 (0.558–1.605)	0.838	0.989 (0.577–1.697)	0.969
High	0.778 (0.452–1.338)	0.364	0.835 (0.485–1.439)	0.516
Smoking				
No	reference		reference	
Yes	0.763 (0.239–2.436)	0.648	0.956 (0.296–3.086)	0.940
Alcohol drinking				
No	reference		reference	
Yes	0.630 (0.360–1.100)	0.104	0.621 (0.337–1.142)	0.125
Sufficient physical activity				
No	reference		reference	
Yes	0.589 (0.374–0.928)	**0.023**	0.636 (0.401–1.009)	0.055
Sufficient sleep				
No	reference		reference	
Yes	0.893 (0.631–1.262)	0.520	0.873 (0.621–1.229)	0.437
Excessive screening time on video				
No	reference		reference	
Yes	1.240 (0.876–1.755)	0.226	1.255 (0.867–1.816)	0.228
Excessive screening time on electronic game				
No	reference		reference	
Yes	1.202 (0.850–1.700)	0.298	1.108 (0.758–1.620)	0.597
Excessive screening time on social media				
No	reference		reference	
Yes	0.998 (0.707–1.411)	0.993	0.953 (0.655–1.386)	0.800
Self-reported obesity				
No	reference		reference	
Yes	0.736 (0.515–1.052)	0.093	0.720 (0.506–1.023)	0.067
MTS-A				
Low	reference		reference	
High	0.775 (0.548–1.097)	0.150	0.822 (0.572–1.181)	0.289
Health literacy level				
Low	reference		reference	
High	0.740 (0.521–1.050)	0.092	0.789 (0.544–1.145)	0.213

*SES* Socioeconomic Status

Sufficient physical activity: Moderate/Vigorous exercise > = 1 h everyday; Sufficient sleep: Sleep at least 8 h; MTS-A: Mental Toughness Scale for Adolescents, the scale [18–72] is composed of six dimensions, including challenge [3–12], commitment [3–12], emotion control [3–12], life control [3–12], confidence in abilities [3–12], and interpersonal confidence [3–12], with a higher score indicating higher mental toughness; Health literacy level: Scores for evaluating personal competencies for the access to, understanding of, appraisal of and application of health information in order to make sound decisions in everyday life

Significant values are shown in bold

**Table 6:** Factors associated with insufficient fruit intake (*N* = 1,423)

	Crude odds ratio (95% CI)	*P*-value	Adjusted odds ratio (95% CI)	*P*-value
Age group				
< 13	reference		reference	
13–14	1.625 (1.135–2.327)	**0.008**	1.497 (1.035–2.166)	**0.032**
> 14	1.575 (1.096–2.264)	**0.014**	1.486 (1.024–2.156)	**0.037**
Gender				
Female	reference		reference	
Male	1.181 (0.855–1.632)	0.313	1.080 (0.753–1.548)	0.676
SES				
Low	reference		reference	
Medium	0.628 (0.361–1.090)	0.098	0.670 (0.384–1.169)	0.159
High	0.330 (0.190–0.571)	**< 0.001**	0.371 (0.213–0.643)	**< 0.001**
Smoking				
No	reference		reference	
Yes	3.422 (0.783–14.962)	0.102	2.494 (0.522–11.907)	0.252
Alcohol drinking				
No	reference		reference	
Yes	1.441 (0.736–2.819)	0.286	1.159 (0.584–2.301)	0.672
Sufficient physical activity				
No	reference		reference	
Yes	0.765 (0.502–1.166)	0.213	0.836 (0.545–1.283)	0.412
Sufficient sleep				
No	reference		reference	
Yes	0.898 (0.650–1.242)	0.516	0.969 (0.694–1.354)	0.854
Excessive screening time on video				
No	reference		reference	
Yes	1.019 (0.735–1.413)	0.910	0.925 (0.654–1.309)	0.659
Excessive screening time on electronic game				
No	reference		reference	
Yes	1.543 (1.111–2.144)	**0.010**	1.435 (1.015–2.028)	**0.041**
Excessive screening time on social media				
No	reference		reference	
Yes	1.215 (0.878–1.682)	0.240	1.146 (0.810–1.622)	0.440
Self-reported obesity				
No	reference		reference	
Yes	0.818 (0.581–1.152)	0.249	0.765 (0.542–1.081)	0.128
MTS-A				
Low	reference		reference	
High	0.767 (0.554–1.062)	0.110	0.934 (0.656–1.330)	0.706
Health literacy level				
Low	reference		reference	
High	0.594 (0.429–0.823)	**0.002**	0.674 (0.479–0.950)	**0.024**

*SES* Socioeconomic Status

Sufficient physical activity: Moderate/Vigorous exercise > = 1 h everyday; Sufficient sleep: Sleep at least 8 h; MTS-A: Mental Toughness Scale for Adolescents, the scale [18–72] is composed of six dimensions, including challenge [3–12], commitment [3–12], emotion control [3–12], life control [3–12], confidence in abilities [3–12], and interpersonal confidence [3–12], with a higher score indicating higher mental toughness; Health literacy level: Scores for evaluating personal competencies for the access to, understanding of, appraisal of and application of health information in order to make sound decisions in everyday life

Significant values are shown in bold

### Not having breakfast daily

In the multivariable model, adolescents who reported excessive screen time on social media (aOR = 1.668, 95% CI: 1.279–2.176, *p* < 0.001) were more likely to skip breakfast. In contrast, those with sufficient sleep duration (aOR = 0.579, 95% CI: 0.447–0.751, *p* < 0.001), higher mental toughness (aOR = 0.692, 95% CI: 0.531–0.901, *p* = 0.006), and higher health literacy (aOR = 0.659, 95% CI: 0.507–0.857, *p* = 0.002) were less likely to skip breakfast, indicating that better sleep, psychological resilience, and the ability to understand and apply health-related information may help maintain regular breakfast habits. (Table 4)

### Insufficient vegetable intake

Notably, no factors were found to be significantly associated with insufficient vegetable intake in the survey-weighted multivariable logistic regression model. (Table 5).

### Insufficient fruit intake

Compared with younger adolescents, those aged 13–14 years (aOR = 1.497, 95% CI: 1.035–2.166, *p* = 0.032) and those aged over 14 (aOR = 1.486, 95% CI: 1.024–2.156, *p* = 0.037) were more likely to consume insufficient fruits, indicating a decline in fruit intake with age. Excessive screen time on electronic games (aOR = 1.435, 95% CI: 1.015–2.028, *p* = 0.041) was also associated with inadequate fruit intake, possibly reflecting lifestyle patterns that favor sedentary behaviors and unhealthy eating. In contrast, adolescents with higher SES (aOR = 0.371, 95% CI: 0.213–0.643, *p* < 0.001) and those with a higher level of health literacy (aOR = 0.674, 95% CI: 0.479–0.950, *p* = 0.024) were less likely to report insufficient fruit. (Table 6)

### Sensitivity analysis

The results of the multivariable logistic regression model, which includes variables with *p* < 0.05 identified from the univariable logistic regression, are presented in Supplementary Tables 4 and 5, providing consistent results with the main findings. Furthermore, to strengthen the robustness of our results, a sensitivity analysis using survey-weighted ordinal logistic regression models was conducted, and the detailed results are presented in Supplementary Tables 6–8, showing similar outcomes to the main findings.

## Discussion

### Major findings

This cross-sectional study of 1,423 participants in Hong Kong identified the prevalence rates of three unhealthy dietary behaviors among Hong Kong adolescents and associated factors. The prevalence of unhealthy dietary behaviors was high, with 51.0% (*n* = 726) of participants not consuming breakfast daily, 86.1% (*n* = 1,225) not meeting the recommended daily vegetable intake, and 83.8% (*n* = 1,193) not meeting the recommended daily fruit intake. Skipping breakfast was more likely among adolescents with excessive social media use, and less likely among those with sufficient sleep, higher mental toughness, and higher health literacy. Insufficient fruit intake was more common among older adolescents and those with excessive gaming time, and less common among those with higher SES and those with a higher level of health literacy, while no factors were found to be associated with insufficient vegetable intake.

### Comparison with previous studies

Unhealthy dietary behaviors among adolescents are a global concern due to their association with adverse long-term health outcomes. According to United Nations Children's Fund (UNICEF), in low- and middle-income countries, 42% of adolescents consume sugary soft drinks daily and 46% eat fast food at least once a week, with even higher prevalence observed in high-income countries (62% and 49%, respectively) [44].A regional study involving nine South-East Asian countries reported that 42% of adolescents had inadequate fruit intake, while 26% had insufficient vegetable intake [45]. In comparison, our study found that over 80% of adolescents in Hong Kong consumed insufficient vegetables and had inadequate fruit intake, suggesting a more severe situation. Previous qualitative studies in Hong Kong attribute this to adolescents’ limited awareness of recommended daily intake, lack of cooking skills, and minimal involvement in meal preparation [46]. These factors may reduce their exposure to vegetables and increase reliance on unhealthy outside food options when parental cooking is unavailable [47].

Excessive screen time, particularly on social media, was associated with increased likelihood of breakfast skipping in our study. This finding is consistent with a Swedish cross-sectional study, where adolescents with high screen time were 1.5 times more likely to skip breakfast (OR = 1.5, 95% CI: 1.1–2.1) [48]. Similarly, excessive screen time on electronic games was associated with lower fruit intake. A Korean web-based survey also found that adolescents with high screen time consumed less fruit and vegetables and more fast food [49]. Screen time may negatively influence dietary habits by reducing physical activity, increasing exposure to unhealthy food marketing, and replacing meals with unhealthy snacks [49], and promoted the development of self-objectification [50]. It is noteworthy that no gender differences in unhealthy dietary behaviors were found in the survey-weighted logistic regression model, which contrasts significantly with previous research [51, 52]. Previous studies have indicated that due to the influence of social and peer norms, particularly regarding appearance and body image [53, 54], adolescent females tend to engage disproportionately in weight-control behaviors and exhibit healthier eating attitudes [54–59]. This discrepancy with previous findings may be attributable to the methodological inconsistencies. As prior studies have indicated, the non-weighted generalized linear model (GLM) logistic regression may mask the actual effects between factors when data come from a complex survey design or unequal weighting [60, 61], which is underscores the need for cautious interpretation of the research findings based on non-representative samples.

Health literacy emerged as a protective factor against breakfast skipping and insufficient fruit intake. A study among Iranian female students found that those with higher health literacy showed more positive attitudes and behaviors toward breakfast consumption [62]. Similarly, several studies have linked higher health literacy with increased fruit and vegetable intake [63–65]. For instance, an Australian study reported that proactive health behaviors, better information appraisal, and greater social support were positively associated with dietary quality, particularly fruit and vegetable consumption [64]. The mechanism underlying the phenomenon may be attributed to a greater ability to engage in proactive health behaviors, which is facilitated by an improved capacity to comprehend health information, resulting from a higher level of health literacy [66]. Furthermore, parental health literacy has been demonstrated to have a close association with adolescent health literacy. Consequently, parents may cultivate healthier family eating habits, as adolescents’ home diets are influenced by parental regulation [67].

Lower SES and older age were associated with lower fruit intake. A UK national survey found that fruit consumption declines steadily from age 7 onward, which may be attributed to increased autonomy in food choices and reduced parental support [68]. A multinational study of 114,558 school-aged children across 28 countries also demonstrated that adolescents from higher-SES backgrounds had significantly higher fruit intake, likely due to greater affordability and accessibility of fresh produce [69].

### Strengths & limitations

There are several strengths of this study. Firstly, a large and diverse sample of adolescents was recruited from 20 secondary schools across Hong Kong, enhancing statistical power and improving the generalizability of the findings. Secondly, unhealthy dietary behaviors were evaluated comprehensively, while potentially relevant factors were also assessed including screen time, health literacy, SES, and physical activity. Thirdly, we employed validated instruments such as the MTS-A and HELMA, which ensured robust measurement of psychological factors. Finally, this study adopted an anonymous survey design, which minimized social desirability bias, particularly for sensitive behaviors like smoking, alcohol drinking, and screen time use. However, several limitations should also be acknowledged. While the findings indicated a potential association between variables and unhealthy dietary habit outcome, it is important to note that the cross-sectional nature of the study prevents us from inferring causality, and longitudinal studies are needed to confirm the directionality of observed associations. All data were self-reported by adolescents, which may introduce recall bias, misinterpretation of questions, or socially desirable responses—particularly given the young age of participants. To address this, explicit instructions and examples were provided within the questionnaire, and the confidentiality of the information provided is emphasized to minimize the potential bias that may occur. Likewise, considering the intermingling of food intake patterns, potential measurement bias might occur when assessing fruit and vegetable consumption. Nonetheless, this study employed additional effort to mitigate such bias by incorporating schematic diagrams of fruit and vegetable portion sizes into the questionnaire, thereby enhancing participants’ understanding and improving the accuracy of self-reported data. Furthermore, while the participating districts may provide valuable insights, the findings may not fully represent the entire population of Hong Kong, particularly the non-participating districts, which could have different demographic and socioeconomic characteristics. This may affect the applicability of our results to the broader population, and we recommend caution when generalizing our findings beyond the studied districts. Finally, other contextual factors known to influence dietary behaviors—such as parental dietary habits, home food availability, school nutrition policies, and peer influences—were not assessed in this study.

### Implications for policy and research

The findings of this study highlight the urgent need for targeted public health campaigns to promote healthy dietary behaviors among adolescents in Hong Kong. Given the high prevalence of insufficient fruit and vegetable intake, school-based nutrition education programs should be strengthened to improve students’ awareness of dietary recommendations and the importance of balanced nutrition. Health literacy promotion, particularly in low socioeconomic groups, may serve as a viable strategy to empower adolescents with the knowledge and skills to make healthier food choices. Moreover, the association between excessive screen time and unhealthy dietary habits suggests that policies regulating screen media use and digital advertising of unhealthy foods—especially on social media platforms—should be considered. Collaborative efforts between schools and families are essential to create supportive environments that facilitate access to nutritious foods and encourage physical activity. We advises policymakers to consider designing interventions that address the factors identified in this study, such as sedentary behavior, with particular emphasis on the role of social support, including parental involvement, to enhance the efficacy of these initiatives.

For future research, longitudinal cohort studies could be conducted to establish causal relationships between lifestyle factors and dietary behaviors. In particular, it would be valuable to examine how screen time, physical activity, and mental health evolve over time and influence dietary patterns during adolescence. Additionally, qualitative studies may offer in-depth insights into adolescents’ perceptions of food, barriers to healthy eating, and family or peer influences on their dietary choices. Expanding the scope of future research to include parental behaviors, home food environments, school food policies, and community-level factors will help to better understand the multi-level determinants of adolescent nutrition.

## Conclusions

The majority of participants reported a concerning prevalence of unhealthy dietary behavior behaviors among Hong Kong adolescents, specifically not having breakfast daily and insufficient intake of fruits and vegetables. Key behavioral, psychosocial, and demographic factors—including screen time, physical activity, health literacy, gender, and socioeconomic status—were found to be significantly associated with these unhealthy dietary behaviors. These findings highlight the importance of developing school-based health education, family engagement strategies, and digital media regulation to address these modifiable risk factors. Future research should prioritize longitudinal and intervention studies to inform the design and implementation of dietary promotion programs tailored to adolescents’ needs and contexts.

## Supplementary Information

Supplementary Material 1.

## Data Availability

The datasets used and/or analysed during the current study are available from the corresponding author on reasonable request.

## Abbreviations

FAS II: Family Affluence Scale II
GLM: Generalized linear model
HELMA: Health Literacy Measure for Adolescents
MTS-A: Mental Toughness Scale for Adolescents
SES: Socioeconomic status
WHO: World Health Organization
