# Supplementary material for: Unhealthy dietary behaviors and associated factors among adolescents: a territory-wide study
Source: BMC Public Health. 2026 Mar 18;26:1370. doi: 10.1186/s12889-026-26935-y (PMC13112767; doi:10.1186/s12889-026-26935-y)
Supplement: Supplementary file 1 — Supplementary Material 1. [file 12889_2026_26935_MOESM1_ESM.docx]

**Supplementary Material**

Supplementary Table 1. Population Indicator of Participating and Non-participating Districts

Supplementary Table 2. Survey-weighted Prevalence of Skip Breakfast, Insufficient Vegetable Intake, and Insufficient Fruit Intake Among Adolescents in Hong Kong

Supplementary Table 3. Comparison Between Participants Exhibiting All Three Unhealthy Habits and Those Exhibiting One or Two Unhealthy Habits

Supplementary Table 4. Factors Associated with Skipping Breakfast Adopting an Univariate Analysis-Based Selection

Supplementary Table 5. Factors Associated with Insufficient Fruit Intake Adopting an Univariate Analysis-Based Selection

Supplementary Table 6. Factors Associated with Skipping Breakfast: Results from Ordinal Logistic Regression Analysis

Supplementary Table 7. Factors Associated with Insufficient Vegetable Consumption: Results from Ordinal Logistic Regression Analysis

Supplementary Table 8. Factors Associated with Insufficient Fruit Consumption: Results from Ordinal Logistic Regression Analysis

Supplementary Figure 1. Distribution of Breakfast Consumption among Participants

Supplementary Figure 2. Distribution of Vegetable Consumption among Participants

Supplementary Figure 3. Distribution of Fruit Consumption among Participants

Supplementary Table 1. Population Indicator of Participating and Non-participating Districts

|  | Total  (n=18) | District Participating (n=11) | Non-participating  (n=7) |
| --- | --- | --- | --- |
| Population Size |  |  |  |
| Grade 1 | 62,248 | 42,521 | 19,727 |
| Grade 2 | 60,818 | 4,1679 | 19,139 |
| Grade 3 | 57,859 | 39,946 | 17,913 |
| Grade 4 | 55,280 | 38,512 | 16,768 |

*Grade: Secondary School

Supplementary Table 2. Survey-weighted Prevalence of Skip Breakfast, Insufficient Vegetable Intake, and Insufficient Fruit Intake Among Adolescents in Hong Kong

|  | Respondents=Y  (n) | Prevalence without weighting (%) | Prevalence with weighting (%) |
| --- | --- | --- | --- |
| Unhealthy dietary habits |  |  |  |
| Skip breakfast | 726 | 51.0% | 53.1% |
| insufficient vegetable intake | 1225 | 86.1% | 85.7% |
| insufficient fruit intake | 1193 | 83.8% | 84.9% |

*Y= participant reported had skip breakfast, insufficient vegetable intake, or insufficient fruit intake.

Supplementary Table 3. Comparison Between Participants Exhibiting All Three Unhealthy Habits and Those Exhibiting One or Two Unhealthy Habits

| Variable | Overall | Exhibit all three outcome variables = No | Exhibit all three outcome variables = Yes | **p** |
| --- | --- | --- | --- | --- |
| **n** | 1423 | 850 (59.7%) | 573 (40.3%) |  |
| **Age group** |  |  |  | **<0.001** |
| <13 years | 604 (42.4%) | 399 (66.1%) | 205 (33.9%) |  |
| 13-14 years | 405 (28.5%) | 225 (55.6%) | 180 (44.4%) |  |
| 14+ years | 414 (29.1%) | 226 (54.6%) | 188 (45.4%) |  |
|  |  |  |  |  |
| **Gender** |  |  |  | **<0.001** |
| Female | 802 (56.4%) | 437 (54.5%) | 365 (45.5%) |  |
| Male | 621 (43.6%) | 413 (66.5%) | 208 (33.5%) |  |
|  |  |  |  |  |
| **FAS Status [0-9]** |  |  |  | 0.09 |
| Low [0-2] | 236 (16.6%) | 136 (57.6%) | 100 (42.4%) |  |
| Mid [3-5] | 697 (49.0%) | 402 (57.7%) | 295 (42.3%) |  |
| High [6-9] | 490 (34.4%) | 312 (63.7%) | 178 (36.3%) |  |
|  |  |  |  |  |
| **Smoking** |  |  |  | 0.595 |
| No | 1400 (98.4%) | 838 (59.9%) | 562 (40.1%) |  |
| Yes | 23 (1.6%) | 12 (52.2%) | 11 (47.8%) |  |
|  |  |  |  |  |
| **Alcohol drinking** |  |  |  | 0.094 |
| No | 1304 (91.6%) | 788 (60.4%) | 516 (39.6%) |  |
| Yes | 119 (8.4%) | 62 (52.1%) | 57 (47.9%) |  |
|  |  |  |  |  |
| **Sufficient physical activity** |  |  |  | **<0.001** |
| No | 1239 (87.1%) | 666 (53.8%) | 573 (46.2%) |  |
| Yes | 184 (12.9%) | 184 (100.0%) | 0 (0.0%) |  |
|  |  |  |  |  |
| **Sufficient sleep** |  |  |  | **<0.001** |
| No | 792 (55.7%) | 415 (52.4%) | 377 (47.6%) |  |
| Yes | 631 (44.3%) | 435 (69.0%) | 196 (31.1%) |  |
|  |  |  |  |  |
| **Excessive screening time on video** |  |  |  | **0.036** |
| No | 595 (41.8%) | 375 (63.0%) | 220 (37.0%) |  |
| Yes | 828 (58.2%) | 475 (57.4%) | 353 (42.6%) |  |
|  |  |  |  |  |
| **Excessive screening time on electronic game** |  |  |  | **<0.001** |
| No | 683 (48.0%) | 456 (66.8%) | 227 (33.2%) |  |
| Yes | 740 (52.0%) | 394 (53.2%) | 346 (46.8%) |  |
|  |  |  |  |  |
| **Excessive screening time on social media** |  |  |  | **<0.001** |
| No | 691 (48.6%) | 459 (66.4%) | 232 (33.6%) |  |
| Yes | 732 (51.4%) | 391 (53.4%) | 341 (46.6%) |  |
|  |  |  |  |  |
| **Self-reported obesity** |  |  |  | 0.015 |
| No | 943 (66.3%) | 585 (62.0%) | 358 (38.0%) |  |
| Yes | 480 (33.7%) | 265 (55.2%) | 215 (44.8%) |  |
|  |  |  |  |  |
| **MTS-A** |  |  |  | **<0.001** |
| Low | 784 (55.1%) | 412 (52.6%) | 372 (47.4%) |  |
| High | 639 (44.9%) | 438 (68.5%) | 201 (31.5%) |  |
|  |  |  |  |  |
| **Health literacy level** |  |  |  | **<0.001** |
| Low | 727 (51.1%) | 388 (53.4%) | 339 (46.6%) |  |
| High | 696 (48.9%) | 462 (66.4%) | 234 (33.6%) |  |

Supplementary Table 4. Factors Associated with Skipping Breakfast Adopting an Univariate Analysis-Based Selection

|  | Adjusted odds ratio (95% CI) | *P-value* |
| --- | --- | --- |
| **Age group** |  |  |
| <13 | reference |  |
| 13-14 | 1.256 (0.950-1.662) | 0.110 |
| >=14 | 1.113 (0.841-1.473) | 0.453 |
| **Gender** |  |  |
| Female | reference |  |
| Male | 0.813 (0.625-1.059) | 0.126 |
| **Smoking** |  |  |
| No | reference |  |
| Yes | 2.116 (0.719-6.222) | 0.173 |
| **Alcohol drinking** |  |  |
| No | reference |  |
| Yes | 1.321 (0.820-2.128) | 0.253 |
| **Sufficient physical activity** |  |  |
| No | reference |  |
| Yes | 0.727 (0.502-1.052) | 0.091 |
| **Sufficient sleep** |  |  |
| No | reference |  |
| Yes | 0.581 (0.448-0.752) | **<0.001** |
| **Excessive screening time on electronic game** |  |  |
| No | reference |  |
| Yes | 1.264 (0.972-1.642) | 0.080 |
| **Excessive screening time on social media** |  |  |
| No | reference |  |
| Yes | 1.621 (1.250-2.101) | **<0.001** |
| **Self-reported obesity** |  |  |
| No | reference |  |
| Yes | 1.143 (0.880-1.485) | 0.315 |
| **MTS-A** |  |  |
| Low | reference |  |
| High | 0.687 (0.527-0.894) | **0.005** |
| **Health literacy level** |  |  |
| Low | reference |  |
| High | 0.652 (0.503-0.846) | **0.001** |

Sufficient physical activity: Moderate/Vigorous exercise >=1 hours everyday; Sufficient sleep: Sleep at least 8 hours; MTS-A: Mental Toughness Scale for Adolescents, the scale [18-72] is composed of six dimensions, including challenge [3-12], commitment [3-12], emotion control [3-12], life control [3-12], confidence in abilities [3-12], and interpersonal confidence [3-12], with a higher score indicating higher mental toughness; Health literacy level: Scores for evaluating personal competencies for the access to, understanding of, appraisal of and application of health information in order to make sound decisions in everyday life.

Supplementary Table 5. Factors Associated with Insufficient Fruit Intake Adopting an Univariate Analysis-Based Selection

|  | Adjusted odds ratio (95% CI) | *P-value* |
| --- | --- | --- |
| **Age group** |  |  |
| <13 | reference |  |
| 13-14 | 1.538 (1.067-2.219) | **0.021** |
| >=14 | 1.533 (1.063-2.212) | **0.022** |
| **FAS Status** |  |  |
| Low | reference |  |
| Medium | 0.673 (0.384-1.178) | 0.165 |
| High | 0.366 (0.209-0.640) | **<0.001** |
| **Excessive screening time on electronic game** |  |  |
| No | reference |  |
| Yes | 1.480 (1.060-2.064) | **0.021** |
| **Health literacy level** |  |  |
| Low | reference |  |
| High | 0.650 (0.465-0.910) | **0.012** |

FAS: family Affluence Scale; Health literacy level: Scores for evaluating personal competencies for the access to, understanding of, appraisal of and application of health information in order to make sound decisions in everyday life.

Supplementary Table 6. Factors Associated with Skipping Breakfast: Results from Ordinal Logistic Regression Analysis

|  | aOR (95% CI) | *P-value* |
| --- | --- | --- |
| **Age group** |  |  |
| <13 | reference |  |
| 13-14 | 1.359 (1.061-1.739) | **0.015** |
| >=14 | 1.224 (0.951-1.575) | 0.117 |
| **Gender** |  |  |
| Female | reference |  |
| Male | 0.772 (0.603-0.988) | **0.040** |
| **FAS Status** |  |  |
| Low | reference |  |
| Medium | 1.139 (0.827-1.568) | 0.427 |
| High | 0.861 (0.607-1.223) | 0.404 |
| **Smoking** |  |  |
| No | reference |  |
| Yes | 2.789 (1.141-6.816) | **0.025** |
| **Alcohol drinking** |  |  |
| No | reference |  |
| Yes | 1.299 (0.884-1.909) | 0.183 |
| **Sufficient physical activity** |  |  |
| No | reference |  |
| Yes | 0.708 (0.502-0.998) | **0.049** |
| **Sufficient sleep** |  |  |
| No | reference |  |
| Yes | 0.617 (0.483-0.789) | **<0.001** |
| **Excessive screening time on video** |  |  |
| No | reference |  |
| Yes | 1.080 (0.855-1.365) | 0.519 |
| **Excessive screening time on electronic game** |  |  |
| No | reference |  |
| Yes | 1.217 (0.944-1.568) | 0.129 |
| **Excessive screening time on social media** |  |  |
| No | reference |  |
| Yes | 1.553 (1.217-1.981) | **<0.001** |
| **Self-reported obesity** |  |  |
| No | reference |  |
| Yes | 1.246 (0.979-1.585) | 0.074 |
| **MTS-A** |  |  |
| Low | reference |  |
| High | 0.726 (0.565-0.934) | **0.013** |
| **Health literacy level** |  |  |
| Low | reference |  |
| High | 0.657 (0.513-0.840) | **<0.001** |

Levels of outcomes: "Every day","5 to 6 days", "3 to 4 days","1 to 2 days","Not having breakfast".

FAS: family Affluence Scale; Sufficient physical activity: Moderate/Vigorous exercise >=1 hours everyday; Sufficient sleep: Sleep at least 8 hours; MTS-A: Mental Toughness Scale for Adolescents, the scale [18-72] is composed of six dimensions, including challenge [3-12], commitment [3-12], emotion control [3-12], life control [3-12], confidence in abilities [3-12], and interpersonal confidence [3-12], with a higher score indicating higher mental toughness; Health literacy level: Scores for evaluating personal competencies for the access to, understanding of, appraisal of and application of health information in order to make sound decisions in everyday life.

Survey-weighted analyses were performed using weights derived from grade-level distributions.

Supplementary Table 7. Factors Associated with Insufficient Vegetable Consumption: Results from Ordinal Logistic Regression Analysis

|  | aOR (95% CI) | *P-value* |
| --- | --- | --- |
| **Age group** |  |  |
| <13 | reference |  |
| 13-14 | 1.271 (1.003-1.611) | **0.047** |
| >=14 | 0.904 (0.714-1.145) | 0.404 |
| **Gender** |  |  |
| Female | reference |  |
| Male | 1.151 (0.919-1.441) | 0.222 |
| **FAS Status** |  |  |
| Low | reference |  |
| Medium | 0.634 (0.456-0.880) | **0.006** |
| High | 0.501 (0.356-0.706) | **<0.001** |
| **Smoking** |  |  |
| No | reference |  |
| Yes | 4.896 (1.448-16.552) | **0.011** |
| **Alcohol drinking** |  |  |
| No | reference |  |
| Yes | 0.808 (0.508-1.285) | 0.368 |
| **Sufficient physical activity** |  |  |
| No | reference |  |
| Yes | 0.671 (0.478-0.942) | **0.021** |
| **Sufficient sleep** |  |  |
| No | reference |  |
| Yes | 0.826 (0.669-1.020) | 0.077 |
| **Excessive screening time on video** |  |  |
| No | reference |  |
| Yes | 1.106 (0.876-1.397) | 0.397 |
| **Excessive screening time on electronic game** |  |  |
| No | reference |  |
| Yes | 1.075 (0.848-1.363) | 0.549 |
| **Excessive screening time on social media** |  |  |
| No | reference |  |
| Yes | 1.156 (0.923-1.447) | 0.207 |
| **Self-reported obesity** |  |  |
| No | reference |  |
| Yes | 0.707 (0.567-0.882) | **0.002** |
| **MTS-A** |  |  |
| Low | reference |  |
| High | 0.743 (0.596-0.927) | **0.009** |
| **Health literacy level** |  |  |
| Low | reference |  |
| High | 0.604 (0.482-0.756) | **<0.001** |

Levels of outcomes: "1.5 or more bowls of vegetables daily", "One to less than 1.5 bowls of vegetables daily", "Half a bowl of vegetables", "Less than half a bowl of vegetables", "Occasionally eat one or two vegetables", "Not at all".

FAS: family Affluence Scale; Sufficient physical activity: Moderate/Vigorous exercise >=1 hours every day; Sufficient sleep: Sleep at least 8 hours; MTS-A: Mental Toughness Scale for Adolescents, the scale [18-72] is composed of six dimensions, including challenge [3-12], commitment [3-12], emotion control [3-12], life control [3-12], confidence in abilities [3-12], and interpersonal confidence [3-12], with a higher score indicating higher mental toughness; Health literacy level: Scores for evaluating personal competencies for the access to, understanding of, appraisal of and application of health information in order to make sound decisions in everyday life.

Survey-weighted analyses were performed using weights derived from grade-level distributions.

Supplementary Table 8. Factors Associated with Insufficient Fruit Consumption: Results from Ordinal Logistic Regression Analysis

|  | aOR (95% CI) | *P-value* |
| --- | --- | --- |
| **Age group** |  |  |
| <13 | reference |  |
| 13-14 | 1.328 (1.036-1.702) | **0.025** |
| >=14 | 1.324 (1.040-1.685) | **0.023** |
| **Gender** |  |  |
| Female | reference |  |
| Male | 1.245 (0.989-1.568) | 0.062 |
| **FAS Status** |  |  |
| Low | reference |  |
| Medium | 0.955 (0.700-1.303) | 0.772 |
| High | 0.510 (0.368-0.705) | **<0.001** |
| **Smoking** |  |  |
| No | reference |  |
| Yes | 11.624 (4.626-29.206) | **<0.001** |
| **Alcohol drinking** |  |  |
| No | reference |  |
| Yes | 1.148 (0.784-1.681) | 0.479 |
| **Sufficient physical activity** |  |  |
| No | reference |  |
| Yes | 0.853 (0.624-1.168) | 0.322 |
| **Sufficient sleep** |  |  |
| No | reference |  |
| Yes | 0.865 (0.692-1.081) | 0.203 |
| **Excessive screening time on video** |  |  |
| No | reference |  |
| Yes | 0.984 (0.779-1.244) | 0.896 |
| **Excessive screening time on electronic game** |  |  |
| No | reference |  |
| Yes | 1.060 (0.842-1.336) | 0.619 |
| **Excessive screening time on social media** |  |  |
| No | reference |  |
| Yes | 1.170 (0.928-1.474) | 0.184 |
| **Self-reported obesity** |  |  |
| No | reference |  |
| Yes | 0.891 (0.709-1.119) | 0.320 |
| **MTS-A** |  |  |
| Low | reference |  |
| High | 0.820 (0.650-1.036) | 0.096 |
| **Health literacy level** |  |  |
| Low | reference |  |
| High | 0.632 (0.504-0.792) | **<0.001** |

Levels of outcomes: "Two or more servings of fruit daily","Half serving of fruit daily","One serving of fruit daily","Occasionally eat once or twice","Not at all".

FAS: family Affluence Scale; Sufficient physical activity: Moderate/Vigorous exercise >=1 hours everyday; Sufficient sleep: Sleep at least 8 hours; MTS-A: Mental Toughness Scale for Adolescents, the scale [18-72] is composed of six dimensions, including challenge [3-12], commitment [3-12], emotion control [3-12], life control [3-12], confidence in abilities [3-12], and interpersonal confidence [3-12], with a higher score indicating higher mental toughness; Health literacy level: Scores for evaluating personal competencies for the access to, understanding of, appraisal of and application of health information in order to make sound decisions in everyday life.

Survey-weighted analyses were performed using weights derived from grade-level distributions.


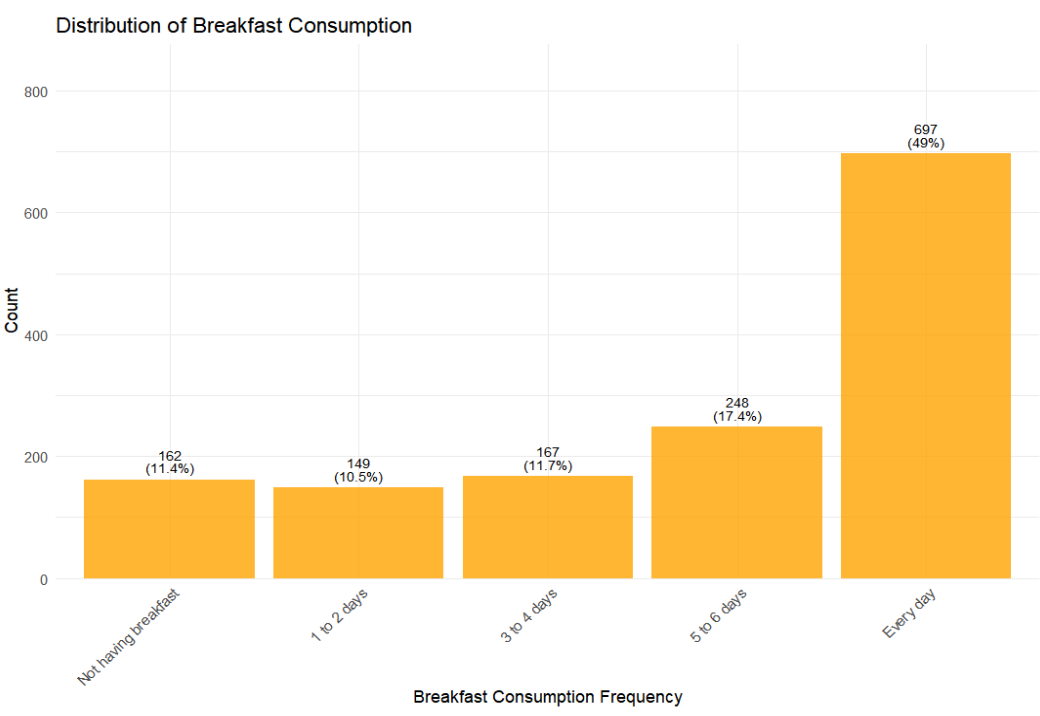


Supplementary Figure 1. Distribution of Breakfast Consumption among Participants


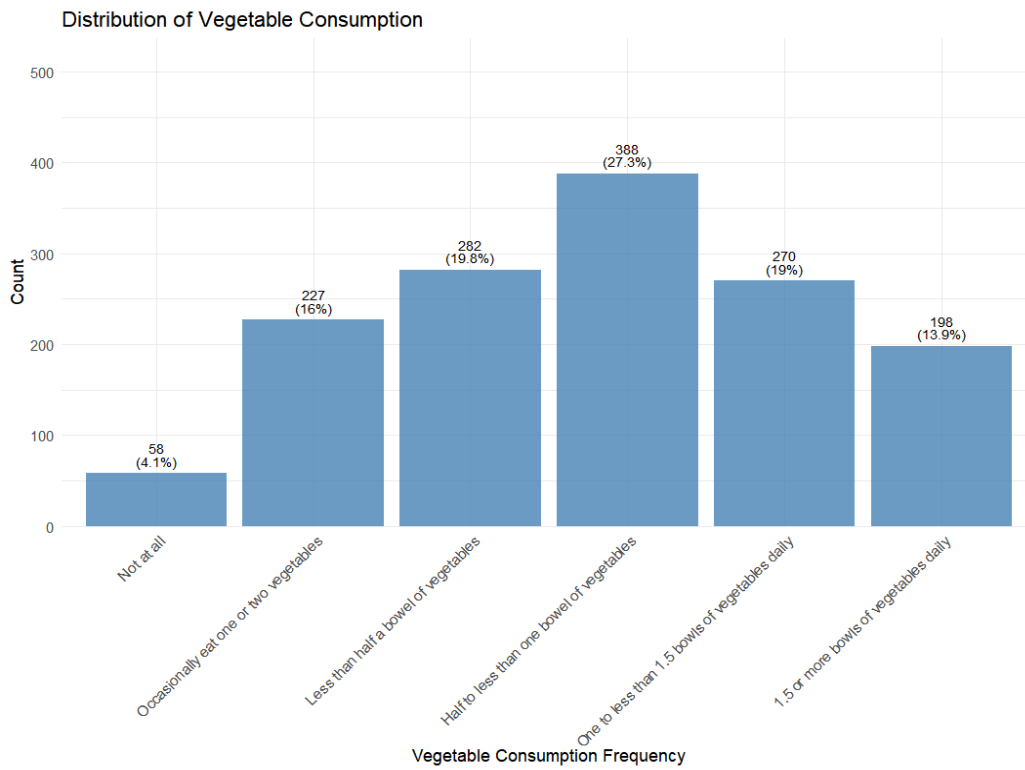


Supplementary Figure 2. Distribution of Vegetable Consumption among Participants


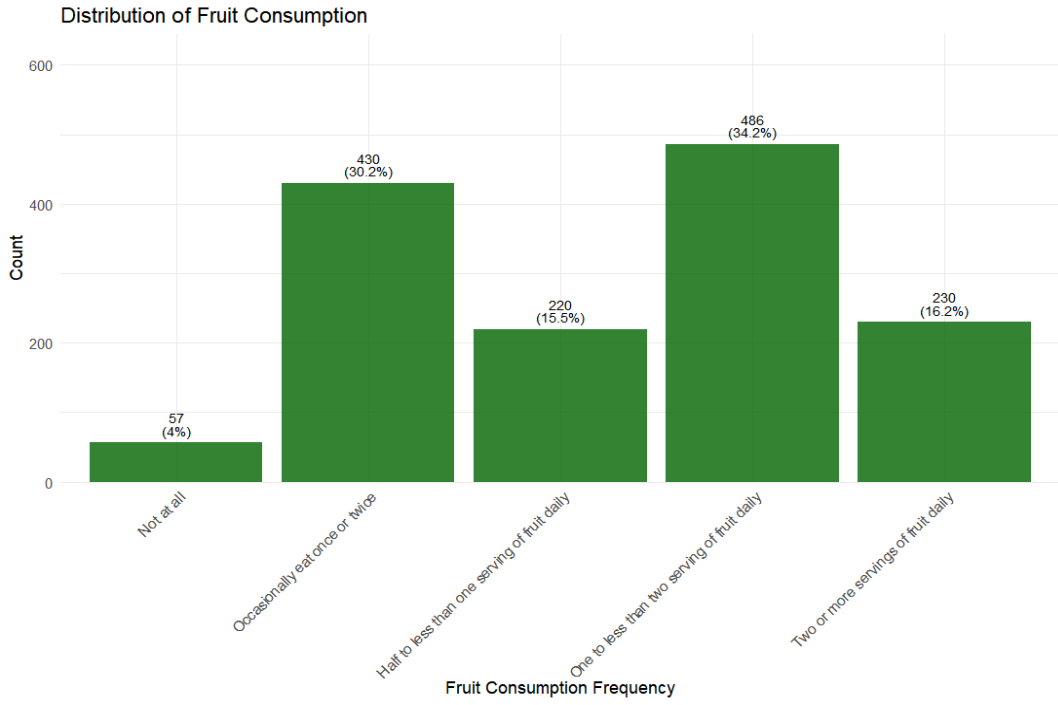


Supplementary Figure 3. Distribution of Fruit Consumption among Participants
